# Dasatinib-Induced Hypopigmentation in Pediatric Patient with Chronic Myeloid Leukemia: A Case Report and Review of the Literature

**DOI:** 10.1155/2018/4062431

**Published:** 2018-07-09

**Authors:** Bader Alharbi, Samer Alamri, Ahmed Mahdi, Siham Marghalani

**Affiliations:** ^1^King Abdullah International Medical Research Center, King Saud bin Abdulaziz University for Health Sciences, P.O. Box 9515, Jeddah 21423, Saudi Arabia; ^2^Department of Dermatology, King Khaled National Guard Hospital, National Guard Health Affairs, P.O. Box 9515, Jeddah 21423, Saudi Arabia

## Abstract

Dasatinib is an oral second-generation multitarget tyrosine-kinase inhibitor (TKI) that is efficacious in treating imatinib-resistant chronic myeloid leukemia (CML) or intolerant cases. Noncutaneous adverse effects with dasatinib are well known in the literature, most commonly cytopenias and fluid retention, while pigmentary abnormalities have rarely been reported. We report the case of a 12-year-old male known case of CML, who presented to dermatology clinic approximately 2 years after initiating dasatinib treatment, with new-onset hypopigmentation of his upper limb, upper chest, and both knees of six months' duration.

## 1. Introduction

Chronic myeloid leukemia (CML) is a hematopoietic stem cell malignancy. It usually occurs in an older population with an age of 60 to 65 years [1]. It is considered to be rare among the young population with an incidence of 2% of all leukemia in an age less than 15 years [2]. CML is a clonal disease that is caused by a gene mutation that consists of a reciprocal translocation between chromosomes 9 and 22, leading to what is known as Philadelphia chromosome (Ph) [3]. Tyrosine kinase inhibitors (TKIs) are currently the mainstay of CML treatment. Dasatinib is an oral multitarget tyrosine-kinase inhibitor. It is efficacious in cases of resistance or intolerance to Imatinib [4]. It works by inhibiting BCR-ABL mutant forms, Src-family tyrosine kinases, c-Kit, ephrin-A2 receptor (EphA2R), and platelet-derived growth factor receptor-B (PDGFR-B). Unlike imatinib, it binds to active and inactive conformations of BCR-ABL [5]. Multiple dermatological side effects such us superficial edema, lichenoid reaction, psoriasis, and Steven-Jonson syndrome have been reported with first generation TKIs like imatinib mesylate [6]. However, depigmentation is reported to be around 41 percent [7]. In contrast, cutaneous side effects of dasatinib have been rarely reported. We report a case of dasatinib-induced hypopigmentation in a young patient with chronic myeloid leukemia and review cases in the literature.

## 2. Case

**Figure 1:**
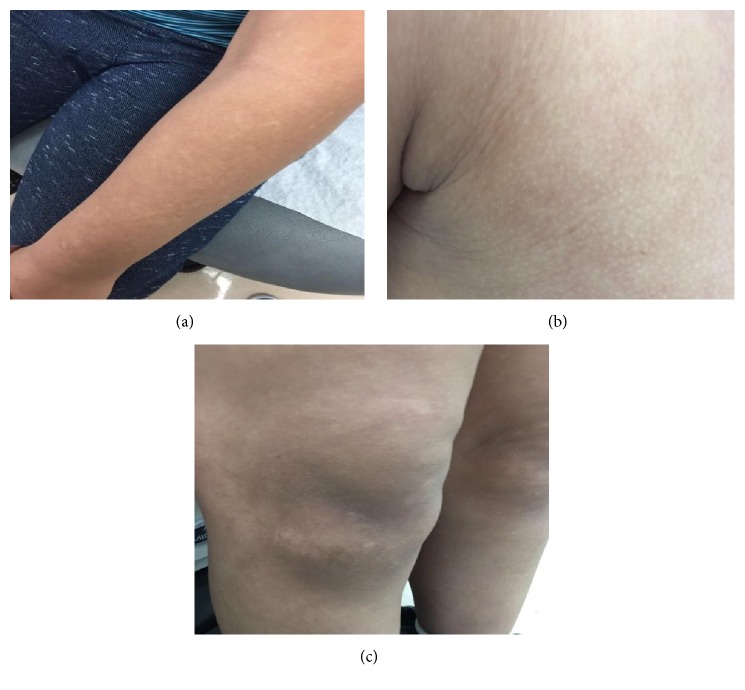
Hypopigmented areas on the (a) upper limb, (b) upper chest, and (c) both knees.

A 12-year-old male with a history of chronic myeloid leukemia presented to our dermatology clinic with new-onset hypopigmented patches that are slowly progressive and of varying sizes of six months' duration on his upper limbs, upper chest, and both knees (Figure 1). Also, two depigmented macules were noted on his upper chest and lower abdomen. The patient denied any rashes or other skin changes and also denied any changes in hair, nail, and mucous membranes. Furthermore, Wood's light examination was negative. The patient was switched to dasatinib, at a dose of 70 mg once per day since two years, due to intolerance to imatinib. There was no personal or family history of autoimmune diseases or pigmentary disorders like vitiligo. The patient denied any use of topical medications or bleaching agents. A 3 mm punch biopsy from active hypopigmented lesion on the abdomen was performed. Histopathologically, it showed decrease melanocytes and basal layer melanin pigmentation. In immunohistochemistry, Melan A stain revealed decreased melanocyte. All positive and negative controls are examined and show appropriate reactivity. The patient was treated with close observation and reassurance. Through it all, the above clinical clues led to a diagnosis of skin depigmentation during dasatinib treatment.

## 3. Discussion

Tyrosine Kinase Inhibitors (TKI) are considered the cornerstone in the treatment of chronic myeloid leukemia (CML). Dasatinib, a second generation TKI, is used as a second line therapy in CML cases where patients are resistant or intolerant to first generation TKI, like imatinib [4]. Likewise, our patient was intolerant to imatinib and switched to dasatinib due to severe bone pain before achieving a complete molecular response. In vitro, dasatinib is considered about 300 times more potent than imatinib; this is due to its ability to bind to both active and inactive conformations of BCR-ABL [5].

Multiple well known cutaneous adverse effects were noted with TKI treatment, for example, superficial edema, maculopapular rash, pigmentary changes, lichenoid reaction, and psoriasiform rash [6]. These side effects were reported particularly with first generation TKI, namely, imatinib, while few cases were reported about the cutaneous side effects with dasatinib. Nevertheless, noncutaneous adverse effects with dasatinib are well known in the literature, most commonly cytopenias and fluid retention [14]. Pigmentary side effects with imatinib were reported in a study done by Arora et al. in which depigmentation and hyperpigmentation were seen in 40.9% and 3.6% of 118 patients, respectively [7].

In addition to BCR-ABL, dasatinib targets multiple tyrosine kinases, such as SCR family kinases, c-Kit, platelet-derived growth factor (PDGFR), and ephrin-A receptor kinases. The protooncogene c-Kit and its ligand stem cell factor (SCF) play a crucial role in the proliferation, migration, and survival of melanocytes. Therefore, inhibiting the c-Kit/SCF signaling pathway is thought to be the reason for pigmentary side effects in a patient receiving TKIs [15]. A clinical example of this signaling pathway is seen in patients with piebaldism, which is an autosomal dominant disorder resulting from a mutation in KIT protooncogene leading to the absence of melanocytes and the appearance of leukoderma on the affected areas [16]. However, dasatinib has a low affinity to c-Kit and PDFGR compared to imatinib, and this is the reason why pigmentary side effects are more pronounced with imatinib compared to dasatinib [6].

The median time for the onset of pigmentary adverse effects is about 2 months after starting TKI therapy (Table 1) [8–13]. Moreover, Webb K et al. reported that a case with dasatinib induced facial depigmentation after almost three years of starting the therapy [8]. In comparison, our patient developed generalized hypopigmentation after about 18 months of switching to dasatinib. The pigmentary changes with TKI appear to be dose-dependent and reversible with a resolution of pigmentary side effects after stopping the treatment [16]. Furthermore, Boudadi et al. reported a case with dasatinib induced hypopigmentation that resolved after stopping the treatment, and then the patient's skin color started to darken beyond her baseline and she experienced diffuse hyperpigmentation. This paradoxical pigmentary changes with TKI were thought to be due to drug-related immune dysregulation in which TKI can act as c-Kit receptor modulator [9].

Regarding the age of patients with dasatinib induced hypopigmentation, our patient is considered the youngest compared to cases reported in the literature (Table 1).

The challenge in the management of pigmentary side effects with TKI is that most patients with hematological malignancies, particularly CML, require the continuation of TKI for long periods even after achieving a complete molecular response. This is because of a high incidence of molecular relapse after withholding the therapy [17]. For this reason, the cessation of dasatinib was not the option in the management for our patient and he was treated with close observation and reassurance.

## 4. Conclusion

Since TKIs are widely used in the treatment of different hematological and nonhematological malignancies, we encourage physicians to take into account the multiple cutaneous side effects that can be caused by this therapy. Additionally, further studies are needed to explore the role of c-Kit/SCF signaling pathway and the role of other factors in the development of pigmentary changes with different TKI generations.

## Figures and Tables

**Table 1:** 

Case number	Age	Gender	Diagnosis	Dasatinib dose	Time to hypopigmentation (Months)	References
Current case	12	Male	chronic myeloid leukemia	70 mg once daily	18	Current case

1	72	Male	chronic myeloid leukemia	100 mg once daily	37	[8]

2	52	Female	Hemangiopericytoma	70 mg twice daily	2	[9]

3	27	Female	chronic myeloid leukemia	100 mg once daily	6	[10]

4	16	Male	Acute lymphoblastic leukemia	100 mg twice daily	1	[11]

5	56	Female	chronic myeloid leukemia	70 once daily	2	[12]

6	29	Female	chronic myeloid leukemia	70 once daily	2	[13]

## Data Availability

Data will be available upon request.
